# Trends of unmet need for family planning among currently married reproductive age women in Ethiopia: A multivariate decomposition analysis

**DOI:** 10.1371/journal.pgph.0000291

**Published:** 2022-09-01

**Authors:** Melkalem Mamuye Azanaw, Dawit Tefera Fentie, Yaynemarnesh Asmare Bukayaw, Ayenew Molla Lakew, Malede Mequanent Sisay

**Affiliations:** 1 Department of Public Health, College of Health Sciences, Debre Tabor University, Debre Tabor, Ethiopia; 2 Amhara Regional Health Bureaus, Zonal Health Office, Gondar, Ethiopia; 3 Amhara Regional Health Bureaus, University of Gondar Referral Hospital, Gondar, Ethiopia; 4 Department of Epidemiology and Biostatistics, Institute of Public Health, College of Medicine and Health Science, University of Gondar, Gondar, Ethiopia; Tata Institute of Social Sciences, INDIA

## Abstract

**Introduction:**

Despite decreasing the percentage of women with unmet needs, Ethiopian women still have a higher unmet need for family planning due to different factors. Therefore, addressing the unmet need for FP provides an opportunity for policymakers in all sectors to respond to the expressed fertility preferences of their population. This study aimed to analyze trends and determinants of changes in unmet needs over time among married women of reproductive age in Ethiopia.

**Methods:**

The study used data from three consecutive Demographic and Health Surveys conducted in Ethiopia (2005, 2011, and 2016). These nationally representative household surveys cover all Ethiopia region and city administrations with population health and other relevant indicators). The study included a total weighted sample of 8642 in 2005, 10204 in 2011, and 9824 in 2016 in the final analysis. Factors contributing to the change in unmet need rate were examined using logit-based multivariate decomposition analysis.

**Results:**

Among married women, unmet needs declined from 33.8% (95% confidence interval (CI):32.8,34.8) in 2005 to 21.0%(95%CI:20.2,21.9) in 2016. In decomposition analysis, the difference in coefficients was responsible for 90% of the overall change in the unmet need rate. In particular, being at the age of 25–49 years, rural place of residence, agrarian regions, and having more than four children were significant predictors of the increase in unmet need rate.

**Conclusions:**

Unmet needs among women have shown a remarkable decline over the last decade in Ethiopia. Policy and program interventions better targeting younger, agrarian regions and rural dwellers would help to maintain a declining trend in unmet needs.

## Introduction

Unmet need for family planning is defined as the percentage of reproductive age women, either married or in a union, who are fertile and sexually active but are not using any types of family planning, and report not needing any more children or wanting to delay having another child [1–4].

In 2017, more than one in ten and five married or in-union women have an unmet need for family planning in the world and Africa, respectively [5, 6]. Despite expected reductions in some countries, the global unmet need for family planning remains around 10% between 2017 and 2030. The greatest dramatic reductions in unmet need for family planning projected to fall to 19% in Africa by 2030. The most dramatic declines in unmet needs are projected to drop from 22% in 2017 to 16% in 2030 in Eastern Africa [5]. As more women use contraceptives, unmet need reduces, and family planning information and services rise to meet demand [3].

The trends in unmet need for family planning differ across sub-Saharan Africa sub-regions. Despite slight improvements in contraceptive prevalence (from 18% to 23%and from 13% to 20%, respectively) in Central and Western Africa between 2000 and 2017, the unmet need remained steady at levels above 20% in 2017(27% in Central Africa and 24% in western Africa). On the other hand, Eastern Africa has drastically deviated from this pattern since 2000, with contraceptive prevalence nearly doubling from 20% to 43% and unmet needs falling from 30% to 22% between 2000 and 2017 [6]. The number of married or in-union women with unmet family planning needs is expected to fall globally, owing to declines in Asia and Europe [7, 8]. Previous decomposition studies indicated that 34% of the overall change in contraceptive use was due to a difference in women’s characteristics with the change in women’s characteristics according to age, educational status, religion, and fertility preference [9]. Moreover, side effects of contraception [10], having three or four children [11, 12], women who have any media exposure [13], an increase in women’s age [10, 14] women with no formal education [12, 14–16], under 18 years old at first marriage, partner with no formal education, and absence of discussion with their partner about family planning [15] were significantly associated with unmet need for family planning using ordinary logistic regression analysis.

According to the current situation analysis of contraception in Ethiopia, over 94% of health institutions provide family planning services. Community awareness, education, and expanding health facilities, are critical for family planning utilization. As a result, the Health Development Army (HDA) is being created to make service utilization easier, and it is a significant component of the government’s health plan, with the purpose of integrating and involving families in order to promote successful practices. They are also involved in promotion and preventive initiatives at the individual and social levels, as well as the frequent facilitation of organized public discourse sessions [2, 17].

Despite a decrease in the percentage of women with unmet needs from 37% in 2000 to 21% in 2016, Ethiopian women still have a higher unmet need for family planning due to different factors [2]. Several studies have been conducted to determine unmet family planning needs’ magnitude and associated factors. However, no study has been conducted on its trends and contributing factors among currently married reproductive age women using decomposition analysis. Addressing the unmet need for FP provides an opportunity for policymakers in all sectors to respond to the expressed fertility preferences of their population while simultaneously improving health, slowing the rate of population growth, and contributing to the achievement of national goals [17].

Based on the rationale mentioned above, this study aimed to assess the trends and determinants of changes in unmet needs over time among married women of reproductive age in Ethiopia based on population-level Demographic and Health survey data.

## Methods and materials

### Study setting

We used secondary data from the Ethiopian EDHS 2005, 2011, and 2016, based on a population-based cross-sectional study. The surveys are nationally representative household surveys that collect data on a wide range of populations, health, and other important indicators across Ethiopia’s nine regions (Tigray, Afar, Amhara, Benishangul-Gumuz, Gambela, Harari, Oromia, Somalia, and Southern Nations Nationalities and Peoples of Region), as well as two city administrations (Addis Ababa and Dire Dawa).

### Data source and measurements

**Fig 1:**
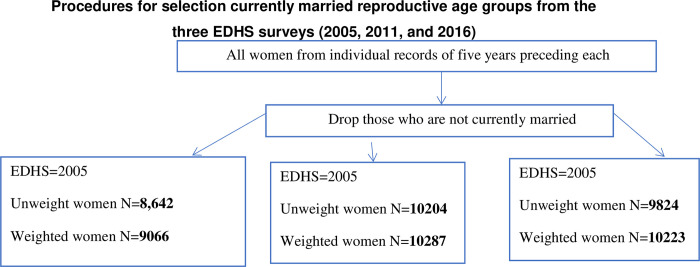
Sample size for unmet need for family planning in Ethiopia based on the EDHS 2005, 2011, and 2016.

The data were accessed from the official database of the DHS program [18] after permission was granted through an online request by explaining the objective of our study. Participants in the survey were asked retrospective questions spanning 5 years before each survey. Our data are restricted to currently married women aged 15–49 years. Based on these criteria, our sample sizes from the three Ethiopian Demographic and Health Surveys (EDHS) were 8642 in 2005, 10204 in 2011, and 9824 women in 2016 (Fig 1).

For each survey year, sample was gathered in two phases. Stratum was also carried out in both urban and rural areas around the country. 540, 624, and 645 EAs were chosen in the first phase in 2005, 2011, and 2016, respectively. In the second phase, systematic sampling was used to select a specified number of households for each EA. The entire sample technique for each survey can be seen in the EDHS results on the Measure DHS website [19].

### Study variables

#### Outcome variables

Unmet need for family planning (Yes, No).

#### Independent variables

Heads of the household(Male, Female), woman’s previous partner(Once, More than once), women educational status(No, Primary, Secondary, Above secondary), husbands educational status(No, Primary, Secondary, Above secondary), current working status(Yes, No), occupation status of the women (No work, Agriculture employee, Non-agriculture work), occupation status of the husband (No work, Agriculture employee, Non-agriculture work, religion(Orthodox, Muslim, Protestant, Tradition/other/catholic), residency(Urban, Rural), wealth index(Richest, Rich, Middle, Poor, Poorest), number of living children(No, One -two, Three-four, More than four), husbands’ desire to children(Wants the same, Wants more, Wants fewer), age at first marriage(15–17, 18–24, more than 24, have knowledge of modern contraceptive methods(Yes, No), visited by family planning worker(Yes, No), visited a health facility (Yes, No), told of family planning at the health facility (Yes, No), and mass media exposure(Yes, No) were independent variables.

*Operational definitions*. Non agriculture employee for women: Percent distribution of women age 15–49 employed in non-agriculture employee during the last 12 months preceding the survey that includes professional/ technical/managerial, Clerical, Sales and services, skilled manual, unskilled manual [20].

Non agriculture employee for women: Percent distribution of men age 15–49 employed in the 12 months preceding the survey by occupation in non-agriculture employee that includes professional/ technical/ managerial, clerical, sales and services, skilled manual, unskilled manual [20]

More than once previous partner: Percentage of currently married women (women who are either legally or formally married or who are living in a consensual union), by the number of other wives that her partner has [20].

### Statistical analysis

Editing, recording, and analysis were performed using STATA 14 Software. The outcome variables with important predictors were extracted from the Ethiopian Demographic and Health Surveys individual data set using STATA 14 software. The data were weighted using cluster number, primary sampling unit, and strata before any statistical analysis to restore the survey’s representativeness. After considering sampling design with calculating SEs, the total sampling distribution reflects the country’s actual population distribution.

Descriptive and trend analysis of unmet needs, examining factors associated with unmet needs, and decomposition of changes in unmet needs were performed. Data from three EDHS surveys were appended into two and formed three data sets (2005–2011, 2011–2016, and 2005–2016 datasets) after extracting explanatory variables for each study phase for decomposition analysis.

This study employed a trend analysis of unmet needs and the decomposition of changes in unmet needs. The descriptive part is presented using tables line graphs and to determine the source of change in unmet needs over the study period, and decomposition analysis was conducted using the mvdcmp command [21]. The trends were examined separately for 2005–2011, 2011–2016, and 2005–2016. Multivariate decomposition analysis is a regression decomposition of the difference in unmet needs between the two surveys. The decomposition analysis aimed to identify the sources of changes in the unmet need rate over the last 11 years. The analysis focused on how unmet need responds to changes in women’s characteristics and how these factors shape differences across surveys conducted at different times. This difference can be attributed to compositional changes between surveys (i.e., differences in characteristics) and changes in effects of the selected explanatory variables (i.e., differences in the coefficients due to changes in population behavior). Hence, the observed difference in unmet needs between different surveys is additively decomposed into a characteristic (or endowments) component and a coefficient (or effects of characteristics) component.

For logistic regression, the Logit or log-odd of unmet need is taken as: 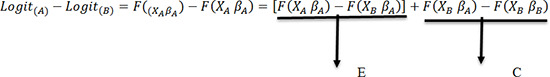


The component labelled ‘E’ refers to the part of the differential attributable to differences in endowments or characteristics (explained component). The ‘C’ component refers to the part of the differential attributable to differences in coefficients or effects (unexplained component) [21].

The equation can be presented as:

Logit(A)−Logit(B)=[β0A−β0B]+ΣXijB*[βijA−βijB]+ΣβijB*[XijA−XijB]

- X_ij_B is the proportion of the j^th^ category of the i^th^ determinant in the DHS 2005,
- X_ij_A is the proportion of the j^th^ category of the i^th^ determinant in DHS 2016,
- β_ij_B is the coefficient of the j^th^ category of the i^th^ determinant in DHS 2005,
- β_ij_A is the coefficient of the j^th^ category of the i^th^ determinant in DHS 2016,
- β_0_B is the intercept in the regression equation fitted to DHS 2005.
- β_0_A is the intercept in the regression equation fitted to DHS 2016.

### Ethics approval and consent to participate

It is not applicable because the data was collected by the performance monitoring and accountability 2020 (PMA2020)/Ethiopia survey project

## Results

### Socio-demographic characteristics of study participants

A total of 8,642, 10,204, and 9,824 currently married women were included in the survey years 2005, 2011, and 2016, respectively. The mean (± SD) age of respondents in the 2005, 2011, and 2016 surveys were 30.7 (±8.6), 30.7(±8.4), and 31.0 (±8.1) years, respectively. Generally, more than half (68%) of the women had no formal education across the three survey years. Between 2005 and 2016, the magnitude of primary educated women a nearly doubled (from 15.5% to 28.3%), whereas the magnitude of secondary or higher educated women climbed slightly. The proportion of women whose partner had no education was 45.8%, while the proportion with secondary or higher education was 16.5% in 2016. In 2016, most respondents (39.0%) were from Oromia, while only 0.2% were from Harar. The proportion of women married between the ages of 15 and 17 increased by 5.3% (from 35.1% in 2005 to 36.7% in 2016), while the proportion of women getting married between the ages of 18 and 24 increased as well by 5.8% (from 26.8% in 2000 to 32.6% in 2016). (Table 1).

**Table 1:** Respondents’ distribution according to demographic and socio-economic variables from 2005-to 2016 in Ethiopia.

Variables		Survey year		
		EDHS-2005(%)	EDHS-2011(%)	EDHS-2016 (%)
Total women (weighted)		9,066	10,287	10,223
Heads of household	Female	8.7	11.7	12.8
	Male	91.3	88.3	87.2
Women previous partner	Once	76.2	77.6	82.5
	More than once	23.8	22.5	17.5
Women educational status	No education	78.2	65.5	61.2
	Primary education	15.5	27.8	28.3
	Secondary education	5.4	3.7	6.4
	Higher education	0.9	3.0	4.1
Husband education status	No education	59.5	48.9	45.8
	Primary education	27.9	39.5	36.9
	Secondary education	10.3	5.9	9.5
	Higher education	2.1	5.0	7.0
Currently working status	No	74.9	64.2	69.1
	Yes	25.1	35.8	30.9
Occupation status of the women	Not working	68.7	43.4	51.6
	Agriculture employee	19.5	28.8	23.4
	Non_ agriculture workers	11.7	27.9	25.0
Occupation status of the Husband	Not working	0.7	0.9	7.9
	Agriculture employee	83.9	75.2	61.9
	Non_ agriculture workers	15.1	23.9	29.0
Religion	Orthodox	42.9	43.6	40.5
	Muslim	37.2	31.0	34.6
	Protestant	16.4	22.5	22.4
	Tradition/other/catholic	3.4	2.8	2.5
Residency	Urban	10.6	17.9	16.2
	Rural	89.4	82.1	83.8
Wealth index	Richest	18.6	20.3	19.1
	Rich	20.1	18.7	20.3
	Middle	21.0	20.3	20.1
	Poor	20.9	20.6	20.3
	Poorest	19.4	20.2	19.1
Total living children	None	6.6	7.8	6.9
	One to two	29.4	31.5	30.9
	Three to four	29.2	27.5	27.7
	More than four	34.8	33.2	34.5
Husbands desire more children compared to women	Wants the same	33.0	41.4	39.2
	Wants more	17.1	24.5	25.9
	Wants fewer	4.8	8.7	7.1
Age at first marriage	Less than15	35.4	29.0	26.0
	15–17	35.1	36.8	36.7
	18–24	26.8	31.1	32.6
	More than 24	2.7	3.1	4.7

key: Non-agricultural employee for husband and women includes professional / technical /managerial, clerical, sales and services, skilled manual, unskilled manual, domestic service. More than once previous partner means having more than one partner in the previous marriage

### Health facility related characteristics

Women’s knowledge of modern contraceptives increased from 87.4% in 2005 to 98.6% in 2016. In 2016, one in three women was visited by a family planning worker in the previous12 months, nearly halves (49%) of the women visited a health facility in the previous 12 months, and out of those who visited a health facility in the last 12 months, 40.4% were told about family planning methods in the last 12 months. There was a considerable increase in women who had been visited by a family planning worker in the last 12 months from 8.2% in 2005 to 29.7% in 2016, and there was a clear pattern in the proportion of currently married women who visited a health facility in the last 12 months which is a 10% increment during the consecutive survey years. Women who were heard about family planning on radio, TV, and in newspaper/magazine (exposed) in the last 12 months has been 25.9% in 2005 and 27.3 in 2016 (Table 2).

**Table 2:** Health facility related characteristics among currently married women in Ethiopia from 2005–2016.

Variables	Survey year		
	EDHS 2005(%)	EDHS- 2011(%)	EDHS- 2016(%)
**Total women (weighted)**	9,066	10,287	10,223
Have knowledge about contraceptive methods	87.5	97.6	98.7
Visited by family planning worker in the preceding 12 months	8.2	19.3	29.7
Visited health facility in the preceding 12 months	29.0	39.0	48.9
Told of family planning in health facility in the preceding 12 months	31.4	27.8	40.4
Exposed to mass media	25.9	34.0	27.3

### Magnitude of unmet need for family planning over time

**Fig 2:**
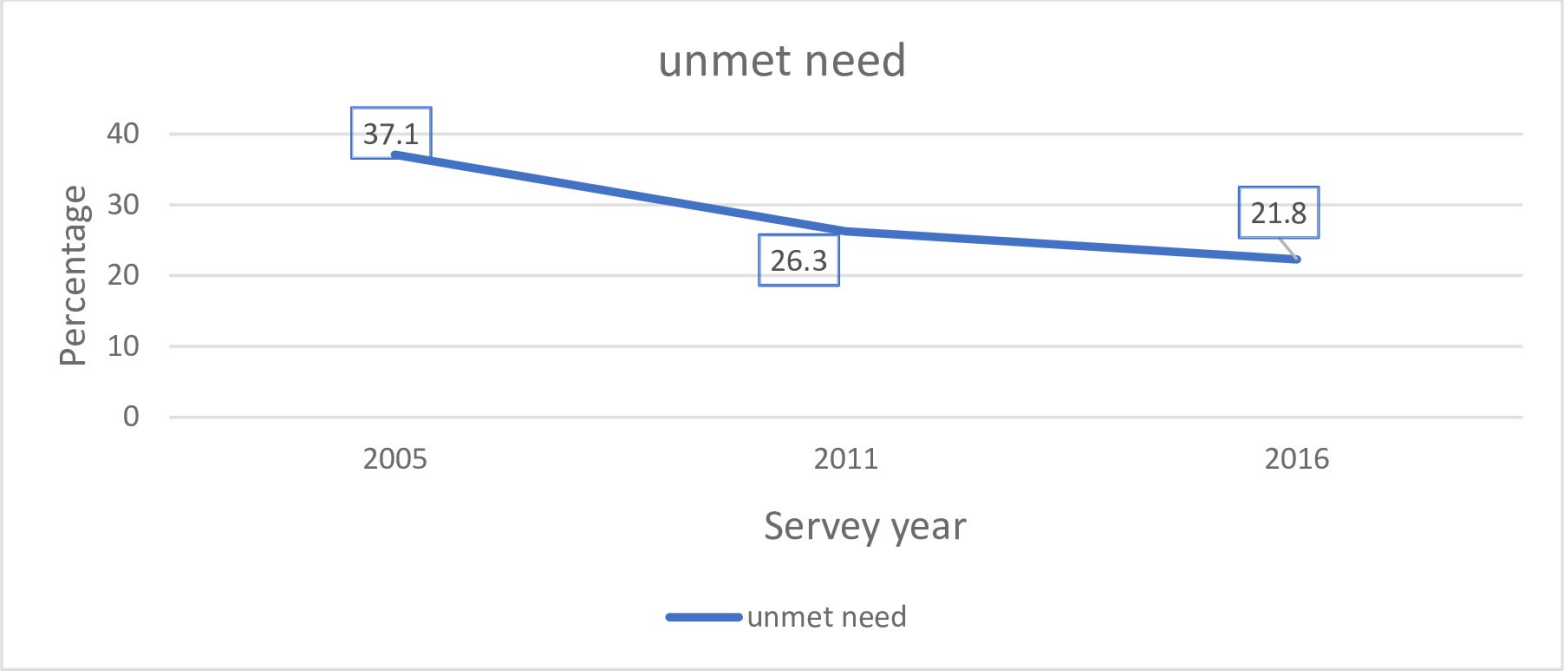
Trends of unmet need for family planning during the three consecutive DHS surveys in Ethiopia.

In Ethiopia, the proportion of unmet need for family planning among currently married women was 33.8% (95%CI: 32.8, 34.8) in 2005, 25.2% (95%CI: 24.4, 26.1) in 2011, and 21.0% (95%CI: 20.2, 21.9) in 2016. The trend period comprises from 2005to 2016 to see the differences in the unmet need rate over time and the potential source for the change in the unmet need rate. The rate of unmet needs over the study period (2005–2016) has significantly declined by 15%. (Fig 2).

#### The trends in unmet need rate by women’s characteristics

**Fig 3:**
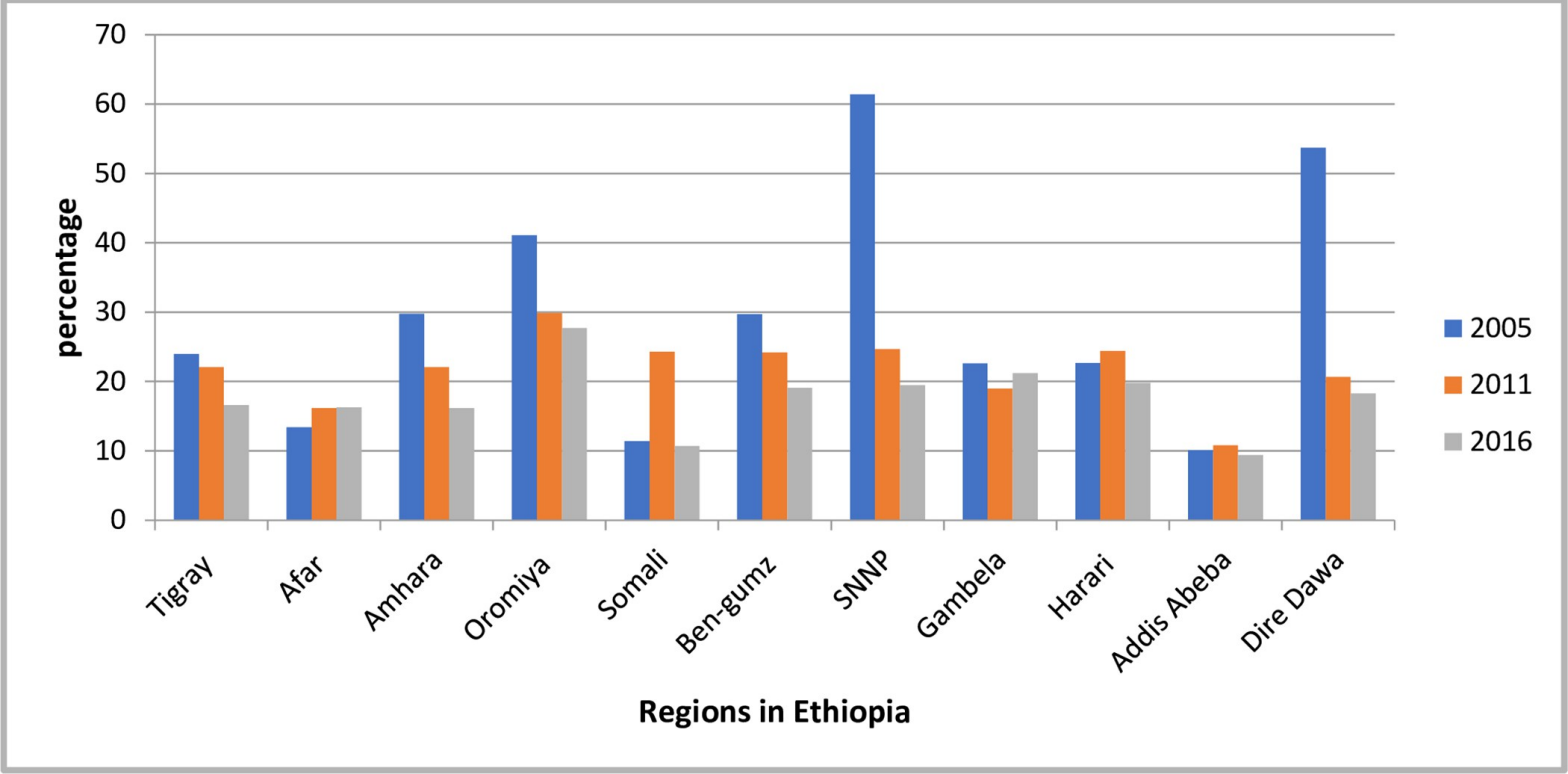
The trend of unmet need for family planning over time across regions in Ethiopia 2005, 2011 and 2016.

The unmet need for family planning among currently married women varied according to their characteristics. During the study period, the percentage of women in rural regions with unmet needs declined. Concerning the region, the unmet need rate decreased in the first phase at 36.7 points in SNNP and declined in the second phase at a 5.2-point (Fig 3). Furthermore, there was a considerable decrease in unmet needs in protestant religious followers by 20.6 points. Over the study period, the proportion of women having an unmet need for family planning decreased by 13.9 points in terms of knowledge of modern contraceptive methods. (Table 3).

**Table 3:** Magnitude of unmet need for family planning among currently married women of reproductive age by selected characteristics in 2005, 2011, and 2016.

Variable		EDHS 2005 N = 3026	EDHS 2011 N = 2554	EDHS 2016 N = 4663	Combined year point difference in unmet need 2016–2005
Heads of household	Female	33.9	26.6	24.4	-9.5
	Male	33.7	25.1	22.9	-10.8
Residency	Urban	16.7	14.9	10.5	-6.2
	Rural	35.7	27.5	23.1	-12.6
Religion	Orthodox	31.2	21.9	16.9	-15.2
	Muslim	34.1	29.7	27.8	-14.3
	Protestant	37.8	25.1	17.2	-20.6
Women previous partner	Once	34.7	26.1	20.8	-13.9
	More than once	30.5	22.3	22.5	-8.0
Age at first marriage in years	Less than 15	33.9	25.0	23.9	-10.0
	15–17	33.2	28.2	21.4	-11.8
	18–24	34.6	22.9	19.1	-15.5
	More than 24	27.9	16.3	16.7	-11.2
Education status of women	No education	34.4	26.2	23.9	-10.5
	Primary education	36.9	26.7	21.4	-15.5
	Secondary education	17.2	12.3	19.1	-1.9
	Higher education	15.6	6.9	16.7	1.1
Educational status of husband	No education	32.5	25.9	23.1	-9.4
	Primary education	39.3	27.2	21.6	-17.7
	Secondary education	29.3	17.5	14.2	-15.1
	Higher education	14.7	13.6	14.9	0.2
Current working status	No	34.0	27.0	21.6	-12.4
	Yes	32.8	22.1	19.9	-12.9
Occupation of the women	Not working	34.3	26.3	23.3	-11.0
	Agriculture employee	35.6	26.7	22.5	-13.1
	Non-agriculture workers	26.9	22.0	19.2	-7.7
Occupation of the Husband	Not working	24.9	26.1	20.5	-4.4
	Agriculture employee	36.1	28.3	23.7	-12.4
	Non-agriculture workers	21.0	15.6	15.0	6.0
Wealth index	Richest	23.8	14.7	23.0	-0.8
	Richer	36.2	26.7	20.0	-16.2
	Middle	36.7	28.2	22.3	-13.9
	Poorer	37.8	26.4	25.8	-12.0
	Poorest	32.9	30.4	24.9	-8.0
Husbands’ desire for the child compared to women	Wants same	31.0	21.9	18.5	-12.5
	Want fewer	33.2	26.3	23.1	-10.1
	Wants more	34.8	24.6	21.5	-13.3
Total children in the household	None	23.5	16.9	13.6	-9.9
	One to two	28.8	19.6	13.2	-15.6
	Three to four	34.8	25.1	20.5	-14.3
	More than four	38.9	32.8	30.0	-8.9
Knowledge of contraceptive method	No	24.2	24.5	13.2	-11.0
	Yes	35.1	25.3	21.2	-13.9
Visited by family planning worker in the last 12 months	No	33.6	25.7	21.6	-12.0
	Yes	34.4	23.6	19.8	-14.6
Visited health facility in the last 12 months	No	34.0	26.0	22.5	-11.5
	Yes	33.0	24.1	19.6	-13.4
Told of family planning at a health facility	No	34.8	25.2	20.3	-14.5
	Yes	29.3	21.3	18.5	-12.4
Media exposure	No	35.0	27.2	22.8	-12.2
	Yes	30.0	21.4	16.3	-13.7

### Decomposition analysis

Overall, from 2005 to 2016, there has been a significant decline in the unmet need rate in Ethiopia. The overall decomposition result revealed that changes in women’s characteristics and the effects of characteristics between surveys explained the decline in unmet needs over time. The difference in the effects of characteristics accounted for almost 90% of the overall change in the unmet need rate, while the remaining 10% was related to changes in women’s characteristics (Table 4).

**Table 4:** Detailed decomposition analysis of change in unmet need for family planning among currently married women in Ethiopia 2005–2016.

Unmet need for family planning		Difference due to characteristics(E)-0.01(-0.02, -0.002)9.7%		Difference due to coefficient (C)-0.12(-0.14, -0.10)90.3%	
		Coefficient (95%CI)	P-value (Percent)	Coefficient (95%CI)	P-value (Percent)
Age in years	15–19	0	0	0	0
	20–24	0.0003(-0.0001,0.001)	0.168(-0.26)	0.004 (-0.011,0.0199)	0.59 (-3.31)
	25–49	**-0.003(-0.005, -0.001)**	0.002(2.23)	0.041 (-0.030,0.112)	0.26(-31.68)
Residency	Urban	0	0	0	0
	Rural	**-0.004(-0.007, -0.002)**	0.001(3.46)	0.017 (-0.066,0.101)	0.69(-13.24)
Region	Urban setting	0	0	0	0
	Agrarian	0.000004(-0.0001,0.0001	0.89(-0.003)	**-0.115 (-0.190,0.041)**	0.002(88.90)
	Emerging	**0.221(0.0008, 0.0018)**	0.001(-0.99)	-0.005(-0.011,0.001)	0.105(3.76)
Religion	Orthodox	0	0	0	0
	Muslim	**0.002(0.001,0.002)**	0.001(-1.26)	**0.029(0.015,0.043)**	0.001(-22.30)
	Protestant	-0.001(-0.002,0.001)	0.325(0.42)	-0.008(-0.018,0.001)	0.087(6.33)
Women previous partner	Once	0	0	0	0
	More than once	-0.002(-0.003,0.0001)	0.059(1.30)	**0.014 (0.003,0.025)**	0.013(-10.94)
Women educational status	No	0	0	0	0
	Primary	0.004(-0.0004,0.0069)	0.280(-2.80)	0.002 (-0.005,0.010)	0.543(-1.77)
	Secondary	0.001(-0.001,0.001)	0.127(-0.39)	0.005(-0.0001,0.0106)	0.055(-4.03)
	Higher	0.221(-0.001, 0.004)	0.233(-1.15)	0.001(-0.001,0.002)	0.446(-0.51)
Age at first marriage in years	Less than15	0	0	0	0
	15–17	-0.0001(-0.0005,0.0003)	0.531(0.10)	-0.002 (0.018,0.013)	0.762(1.88)
	18–24	0.001(-0.001,0.002)	0.542(-0.41)	-0.009(-0.0226,0.0041)	0.174(7.14)
	Greater than 24	0.0002(-0.001,0.001)	-0.17(0.71)	-0.0008(0.0038,0.0022)	0.587(0.64)
Current working status	No	0	0	0	0
	Yes	0.001(-0.001,0.002)	0.379(-0.57)	0.002(-0.009, 0.012)	0.757(-1.23)
Occupation status of husband	Not working	0	0	0	0
	Agricultural employee	-0.005(-0.014,0.004)	0.293(3.66)	-0.02(-0.158,0.114)	0.753(16.84)
	Nonagricultural worker	-0.001(-0.007,0.006)	0.834(0.54)	-0.003(-0.027,0.021)	0.820(2.18)
Total living children	None	0	0	0	0
	One to two	0.0004(-0.0003,0.0011)	0.248(-0.31)	-0.01(-0.039, 0.014)	0.342(9.87)
	Three to four	**-0.002(-0.003,0.001)**	0.001(1.52)	-0.006(-0.035,0.023)	0.694(4.53)
	More than four	**0.0003(0.0001,0.0005)**	0.001(-0.01)	0.01(-0.021,0.049)	0.442(-10.64)
Visited by HW	No	0	0	0	0
	Yes	-0.003 (-0.008,0.003)	0.325(2.06)	-0.001(-0.006,0.003)	0.495(1.15)
Visit HF	No	0	0	0	0
	Yes	0.0020(-0.007,0.002)	0.307(1.79)	-0.005(-0.017,0.006)	0.350(4.20)
Media exposure	No	0	0	0	0
	Yes	-0.0001(-0.0006,0.0005)	0.824(0.04)	-0.0001(-0.013,0.012)	0.981(0.11)
Constant				-0.06(-0.265,0.147)	0.576(45.36)

Abbreviations: HW = health worker; HF = health facility

The age of the respondents, place of residence, region, religion, and the number of total living children are variables that contributed a lot in the change in unmet need for family planning. A decline in the proportion of women under the age category between 25to 49 years showed a significant positive contribution to the unmet need for family planning rate. An increase in the percentage of emerging/pastoralist residents, on the other hand, had a considerable negative influence on the magnitude of unmet demand for family planning. An increased in the composition of women who had more than four living children had slightly decreased unmet need rate, whereas a decrease in the proportion of women from 29.2% to 27.7% (Table 1) who had three to four living children over time increases the rate of unmet need by 1.52% (Table 4) among currently married reproductive age group women. The unmet need rate increased by 3.46% (Table 4) as the proportion of women in rural areas declined from 89.4% to 83.8% (Table 1). Although the change was small (1.26%) (Table 4), a drop in the proportion of Muslim religious followers in the sample from 37.2% to 34.6% (Table 1) revealed a significant decrease in unmet family planning needs.

After controlling for the role of compositional changes, 90% of the decline in unmet need rate was due to the difference in the effects of characteristics. Factors that significantly affected the observed change in unmet need rate were women’s previous partner, region, and religion. (Table 4). Other factors being equal, about 90% of the increase in unmet need for family planning was due to a change in unmet need behaviors among the agrarian population. Compared with Orthodox Christian religious followers, Muslim religious followers showed a 22.3% contribution to the observed decline in unmet need for family planning. Compared to women who had only one previous partner, those who had more than one previous partner had a 10% decline on the unmet need for family planning over the last decades (Table 4).

## Discussion

The study aimed to examine the trends and the major contributing factors that possibly change unmet need rates positively or negatively among currently married reproductive age group women in Ethiopia over the last 11 years. The study revealed that trends in the unmet need have been significantly declining over time (2005–2016). This finding is consistent with the study done in Bangladesh, Pakistan, and Nigeria [14, 22, 23]. This might be attributed to establishing health extension packages in Ethiopia that incorporate family planning packages and a health development army in 2010 to facilitate access to essential maternal health services, especially for rural residents [17]. The current finding is also lower than with the study conducted in developing countries including sub-Saharan, south Asia, and western Asia) [24] and study on the National, regional, and global rates and trends in unmet need for family planning between 1990 and 2015 [25]. This decline might be attributed to the present expansion of health facilities and increased access to health services, such as family planning services, in Ethiopia and the efforts by both governmental and non-governmental organizations [26].

Changes in women’s characteristics accounted for just 10% of the change in the unmet need rate among married reproductive age group women over the last eleven years. The finding is much lower than the previous reports in Ethiopia [27] and a study in Cameroon [28]. This indicated that the significant contribution of change arises when the composition of the population changes according to essential variables. The composition changes in women’s age, place of residence, region, religion, and number of living children significantly affected the change in the unmet need for family planning.

A decrease in the composition of women’s age group of 25–49 years showed a significant positive effect on the unmet need rate as compared to women less than 20 years age group. This finding supports previous studies done in different countries [10, 14, 28, 29]. This finding can explain that when age increases, the exposure to the different family discussions might also increase, which ultimately increases people’s awareness of maternal health service utilization [29].

The effect of religion has become more important over time. Compositional changes in women’s religion were negatively and positively associated with a trend in the unmet need for family planning. This finding was supported by previous studies [9, 23]. A decrease in the proportion of Catholics/tradition/other religions (more unmet need than Orthodox Christian) negatively affected the trends in the unmet need rate. The finding implies a decrease in the proportion of samples taken from women with Catholics/tradition/other religions affected to increase the unmet need for family planning compared to Orthodox Christian followers. The higher unmet need among Orthodox Christian religious followers might be due to more regular attendance at religious services or the influences of client perceptions towards family planning [30, 31].

A decrease in the composition of women with a rural residence in the sample showed a significant effect on the decline in the unmet need for family planning compared to urban residences. This finding is consistent with previous studies in Ethiopia [27], and Government commitments to improve family planning and other health service access might explain this finding. As noted in previous studies [11, 17], the implementation of the Health Extension Program is the major reason, with the provision of family planning services in the rural areas and recently in urban settings of the country. For this purpose, more than 38,000 Health Extension Workers were deployed, mostly in the rural parts of the country. Family planning, one of the 17 packages of rural health care provided by Health Extension Workers, has its objectives to address misconceptions concerning family planning in the community, counsel clients on all family planning methods, and provide short-acting modern contraceptive methods [17]. Moreover, this might be due to an increase in urban residents over time due to in-migration from rural residences and increased effective implementation of health extension packages and active health development army in rural residences than urbanism in family planning implementation. Therefore, urban health extension packages should be expanded through a combined community engagement to strengthen family planning services.

An increased proportion of the sample taken from emerging regions had a negative effect on the unmet need for family planning among the married reproductive age group. This might be due to an increased flow of people from the emerging region to an urban setting to have a standard of living [32].

Furthermore, compared to women without children, women with living children three to four children had a significant negative effect on the change in unmet need for family planning, whereas women with children more than four children had a very small negative effect on unmet need for family planning. This might be due to women who have more than three children who have different exposure and knowledge towards family planning utilization. The finding supports the studies done in India, Burkina Faso, and Nigeria [11, 13, 22].

Almost all of the decline in the trend of unmet need for family planning over the past decade was due to the difference in the effects of characteristics (coefficients). This finding is supported by studies in Ethiopia and Rwanda, where most of the decline in contraceptive use was due to a change in coefficients [9, 33].

One of the findings from the decomposition analysis was the effect of regions. About 88.9% of the increase in unmet need for family planning among currently married reproductive age women in the past decade was due to changes in the agrarian population’s need for family planning behavior. An increase in the unmet need for family planning during the past decade is significantly associated with a coefficient change in the agrarian region compared to urban regions. Agrarian women’s prevalence of unmet need for family planning (64%) far exceeds urban setting prevalence (13%), so the most significant increase has been in the Agrarian areas, where most of the population lives. The Health Extension Program should be strengthened with the provision of family planning services in the agrarian areas to satisfy family planning needs.

Another remarkable finding in this part of the analysis was the effect of religion. About one-fourth of the decline in unmet need for family planning among currently married reproductive age group women for the last 11 years was due to the change in behaviors of women’s religion. The changes in unmet need for family planning differ significantly among the categories of religion. One-fourth of the decline in unmet need for family planning since 2005 has been among followers of the Muslim religions. As recognized in previous studies [9], the effect of religion has become more important over time. Although there is no supporting evidence on the reasons for the difference among religions, religious belief is one of the barriers when women think about using a method for fertility regulation. However, studies may need to understand the major reasons for slow progress in adopting family planning to identify factors with programmatic relevance.

The last remarkable finding in this part of the analysis was the effect number of unions. The unmet need for family planning among women currently married reproductive age group for the last 10 years differed in the number of unions due to the change in behaviors of women’s previous number of partners. A decrease in unmet need for family planning was due to small changes in women who had more than once the number of unions compared to once unions. This might be because when women have a different number of partners, the awareness and exposure to the family planning method are also increased.

### Strength and limitations of the study

To the best of our knowledge, this study is the first of its kind that identifies the trend contributions of factors to the change in unmet need for family planning in Ethiopia. The study utilized a large dataset representing the whole country, Ethiopia. Large sample size with weighting the data for the sampling probability and non-response to make the data nationally represented. Complex sampling procedures were also considered during testing of statistical significance. However, since the data was collected based on self-report from mothers, this could make the data prone to recall and social desirability bias. Furthermore, the current study has limitations related to data and analysis. In particular, the study didn’t show cause effect relationship. The study also has a limitation related to decomposition methods, and sensitivity analysis.

## Conclusions

The unmet need for family planning among women of the reproductive age group in Ethiopia has shown a significant decline over the last 11 years. The majority of the overall change in unmet need rate over the study period was attributable to the change in coefficients of selected explanatory variables between 2005 and 2016.

Only 10% of the change in unmet need for family planning among currently married reproductive age group women in the past decade was due to the change in women’s characteristics. The change in the composition of women’s age, place of residence, region, religion, and the number of living children showed a significant effect on the change in unmet need for family planning, and also the remaining an overall change in unmet need was due to change in coefficients. So that during all phases of decomposition analysis, the variables consistently associated with the change in unmet need for family planning due to characteristics and due to coefficients were the age of the respondents, residence, region category, religion, number of living children, and number of unions.

Program interventions should be targeted areas with low family planning services, especially for some population categories, including rural residing women, younger (adolescents), and Orthodox Christian followers. Therefore, programmers and policymakers shall strengthen their efforts to develop strategies that can meet young women’s need for family planning and advance it further.

## Supporting information

S1 Data (DTA)
